# Transcriptome Analysis of *Pseudomonas aeruginosa* Cultured in Human Burn Wound Exudates

**DOI:** 10.3389/fcimb.2018.00039

**Published:** 2018-02-27

**Authors:** Manuel R. Gonzalez, Verena Ducret, Sara Leoni, Betty Fleuchot, Paris Jafari, Wassim Raffoul, Lee A. Applegate, Yok-Ai Que, Karl Perron

**Affiliations:** ^1^Microbiology Unit, Department of Botany and Plant Biology, Sciences III, University of Geneva, Geneva, Switzerland; ^2^Plastic, Reconstructive and Hand Surgery, Unit of Regenerative Therapy, Centre Hospitalier Universitaire Vaudois, Lausanne, Switzerland; ^3^Department of Intensive Care Medicine, Bern University Hospital, Bern, Switzerland; ^4^School of Pharmaceutical Sciences, University of Geneva and Centre Hospitalier Universitaire Vaudois, Geneva, Switzerland

**Keywords:** *Pseudomonas aeruginosa*, human burn wound exudates, infection, virulence factors, transcriptome

## Abstract

*Pseudomonas aeruginosa* is a severe opportunistic pathogen and is one of the major causes of hard to treat burn wound infections. Herein we have used an RNA-seq transcriptomic approach to study the behavior of *P. aeruginosa* PAO1 growing directly on human burn wound exudate. A chemical analysis of compounds used by this bacterium, coupled with kinetics expression of central genes has allowed us to obtain a global view of *P. aeruginosa* physiological and metabolic changes occurring while growing on human burn wound exudate. In addition to the numerous virulence factors and their secretion systems, we have found that all iron acquisition mechanisms were overexpressed. Deletion and complementation with pyoverdine demonstrated that iron availability was a major limiting factor in burn wound exudate. The quorum sensing systems, known to be important for the virulence of *P. aeruginosa*, although moderately induced, were activated even at low cell density. Analysis of bacterial metabolism emphasized importance of lactate, lipid and collagen degradation pathways. Overall, this work allowed to designate, for the first time, a global view of *P. aeruginosa* characteristics while growing in human burn wound exudate and highlight the possible therapeutic approaches to combat *P. aeruginosa* burn wound infections.

## Introduction

*Pseudomonas aeruginosa* is a major opportunistic Gram-negative bacteria responsible for life-threatening infections in critically ill or immune-compromised patients (Church et al., 2006), and is among the major cause of sepsis after burn trauma (Church et al., 2006). *P. aeruginosa* pathogenicity relies on the production and secretion of a wide range of virulence factors in response to host environments and complex regulatory pathways—including quorum sensing signaling—allowing the bacterial physiology to constantly adapt to changing conditions (Filloux, 2011). Human burn wound is a complex environment composed of necrotic tissue and plasma-derived exudate. We previously reported that, in contrast to other burn wound pathogens such as *Staphylococcus aureus, Acinetobacter baumannii*, or *Escherichia coli*, clinical and laboratory *P. aeruginosa* strains are able to develop in burn wound exudates (BWE) (Gonzalez et al., 2016). Interestingly, while growing in these environments we observed that *P. aeruginosa* strains strongly increased their production of virulence factors including cytotoxic pigment pyocyanin, secreted elastase, or rhamnolipids surfactants (Gonzalez et al., 2016). This occurred however without strong activation of known quorum sensing signaling systems. Moreover, the amount of biofilm produced remained low (Gonzalez et al., 2016).

Several transcriptomics studies on *P. aeruginosa* rodent burn wounds infection models showed clear activation of general pathways involved in iron acquisition and quorum sensing signaling events as well as remodeling of general metabolism (Bielecki et al., 2011; Turner et al., 2014). Due to major differences between human and rodent burn wound characteristics and healing processes (Reviewed in Abdullahi et al., 2014); we decided to investigate the *P. aeruginosa* PAO1 genome expression profiles while growing on human BWE directly collected at wound sites. The transcriptomic approach could provide a detailed view of bacterium adaptation and behavior in the context of burn infections and may contribute in the comprehension of how *P. aeruginosa* infections impact and interfere with wound healing processes. This may also help to better understand host-microbe interactions in a context of human burn wound infections where information remains very limited yet. We hypothesis that the capacity to proliferate in human BWE observed for *P. aeruginosa* may be the consequence of the activation of specific pathways that might be highlighted by gene expression analyses. These pathway identification and analysis of their combination represent essential information in the perspective of finding new potential therapeutic targets specific to burn wound management.

In this study, we have performed a transcriptomic analysis of *P. aeruginosa* in human burn wound exudate coupled with a chemical analysis of BWE composition after bacteria growth, which highlighted the physiological modifications associated with bacterial pathways up-regulation. In addition, an overtime monitoring of gene expression allowed us to identify fine variations in the kinetics of metabolic pathways activation. Altogether, our data highlighted a large remodeling of genome transcription characterized by an induction of several genes involved in pathogenesis, iron acquisition and metabolic adaptation to BWE growth conditions. Interestingly, activation of Quorum Sensing pathways appeared at lower cell density than in standard media suggesting a regulation of bacterial communication characteristic of complex and heterogeneous environments. The results presented in this study provide a detailed understanding of *P. aeruginosa* metabolism and gene expression profile in human burn wound exudate and represents an essential step for the improvement and the development of novel and efficient strategies against *P. aeruginosa* infections and particularly for severe burns.

## Materials and methods

### Bacteria culture conditions

The bacterial strains used in this study are listed in Supplementary Table 1A. *E. coli* and *P. aeruginosa* PAO1 strains were cultured at 37°C in Luria-Bertani (LB) medium (US biological), adjusted to pH 7.0 and pH 9.0, or human burn wound exudate (BWE), collected as described in Baudoin et al. (2016). For experiments, overnight culture were diluted to OD_600_ of 0.05 in fresh medium and transferred in 96-well plates containing 200 μl medium per well. Bacteria were incubated at 37°C with agitation. For complementation experiment pyoverdine produced by *P. protegens* (Sigma-Aldrich) was used.

### DNA manipulations and mutant strain construction

Chromosomal gene deletion was performed by homologous recombination. Briefly, for each gene of interest, two PCR amplification fragments (part A & B) were produced and assembled using the High Fidelity DNA assembly protocol (Gibson Assembly NEB). The fragment was then digested with restriction enzyme BamH1, ligated into the suicide plasmid pME3087 and transformed into *E. coli* DH5α strain by heat-shock using standard methods (Sambrook and Maniatis, 2001). After amplification, extraction, and purification, the suicide plasmid was transformed into *P. aeruginosa* by electroporation (Choi et al., 2006). After enrichment steps, positive deletion events were verified by PCR and sequencing. Primers used to generate *P. aeruginosa* deletion mutants are listed in Supplementary Table 1B. Deleted regions correspond in *hasA* mutant to Δ(3814055…3814574) and in *prrF* mutant to Δ(5284029…5284296). Sequence coordinates refer to the *P. aeruginosa* PAO1 complete genome annotations that are available on Pseudomonas Genome Database (www.pseudomonas.com).

For *prrF* complementation, *P. aeruginosa prrF1/F2* region was amplified by PCR (primers sequences are listed in Supplemental Table 1C), digested with BamH1 and HindIII enzymes and ligated into the pME6001 vector. Plasmids were introduced into *E. coli* TOP10 by heat-shock (Sambrook and Maniatis, 2001), verified by sequencing and then transformed into *P. aeruginosa* by electroporation (Choi et al., 2006).

### Human burn wound exudate collection and chemical analysis

BWE and plasma from burn patients were collected at the Burn Care Unit of the Lausanne University Hospital (CHUV) according to State Ethics Commission for human research (protocol 488/13), as described previously (Gonzalez et al., 2016). The chemical analyses of BWE were performed at the Laboratory of Clinical Chemistry (LCC) at the CHUV as mentioned in (Gonzalez et al., 2016). Briefly, human BWE was composed of Cl^−^ (114.27 mM), Ca^2+^ (1.19 mM), K^+^ (4.78 mM), Mg^2+^ (0.90 mM), ${\text{NH}}_{4}^{+}$ (116.00 μM), ${\text{PO}}_{4}^{3-}$ (0.90 mM), iron (5.42 μM), copper (4.71 μM), zinc (14.93 μM), cholesterol (0.59 mM), triglycerides (0.24 mM), proteins (23.40 g/L), urea (10.51 mM), glucose (5.85 mM), pyruvate (175.33 μM), L-lactate (3.19 mM), and the following amino acids in μM concentrations: Ala (342), Arg (18), Asn (47), Asp (21), Cyt (64), Glu (65), Gln (446), Gly (276), His (85), Ile (64), Leu (152), Lys (177), Met (30), Orn (128), Phe (122), Pro (179), Ser (133), Thr (118), Tyr (99), Val (235). Quantification of Hyp and Pro amino acids was measured by HPLC at the LCC.

### RNA extraction and reverse transcription

RNA extraction was performed as previously described (Gonzalez et al., 2016). Briefly, three independent *P. aeruginosa* cultures were grown in 200 μl medium at either an OD_600_ of 2.0 for the RNA-Seq analysis, or OD_600_ of 0.8, 1.1, 2.2, and 4.6 for gene expression kinetics analysis. These OD_600_ values correspond in BWE conditions to 6, 12, 18, and 24 h of culture and in LB pH 7.0 conditions to 2, 2.5, 3, and 10 h of culture. Each replicate was constituted by a pool of 3 × 200 μl culture, and treated with 1.2 ml RNA Protect bacterial solution (Qiagen), following 10 min incubation at room temperature. Bacteria suspensions were centrifuged, supernatants discarded and pellets stored at −80°C. Total RNA was further extracted using an RNeasy column (Qiagen) according to manufacturers' instructions. Purified RNA was eluted in 50 μl RNAse-free water, and the concentration was quantified using a Qubit fluorometer (Life Technologies). Total RNA was stored at −80°C prior further analysis. Synthesis of cDNA was performed using random hexamer primers (Promega) and Improm-II reverse transcriptase (Promega) according to described protocol (Gonzalez et al., 2016).

### Quantitative RT-PCR

qRT-PCR procedures were performed in duplicate starting from three independent experiments, using SYBR Green mix (Power SYBR Green PCR Master Mix, Thermo Fisher Scientific), according to the manufacturer's instructions. Primers used for qRT-PCR are listed in Supplementary Table 2. Results were analyzed according to the previously described method (Schmittgen and Livak, 2008) using *oprF* (PA1777) gene as an internal control. Analysis was performed in duplicate in three independent experiments and standard deviations are indicated.

### RNA-seq sequencing and bioinformatics analyses

RNA-Seq sequencing was performed at the iGE3 Genomic Platform at University of Geneva medical school (CMU). The ribosomal RNA was removed with the Illumina Ribo-Zero rRNA Removal kit (Bacteria) and the RNA libraries were prepared for sequencing using the Illumina TruSeq mRNA stranded LT protocol according to the manufacturer instructions. The 100 nt long reads were mapped with BWA v.0.7.10 (bwa mem) to the *P. aeruginosa* PA01 RefSeq genome (NC_002516). The gene features were counted with HTseq v0.6.1 and the differential expression analysis was performed with the R/Bioconductor EdgeR v3.4.2 package with a GLM (generalized linear model) test. Gene identity numbers were obtained from Pseudomonas Genome Database (www.pseudomonas.com).

### Statistical analysis

Analyses were performed using three technical replicates in three independent experiments. All data were analyzed for statistical significance using the *t*-test and standard deviations.

## Results

### *P. aeruginosa* gene transcription in burn wound exudate growth conditions

**Figure 1:**
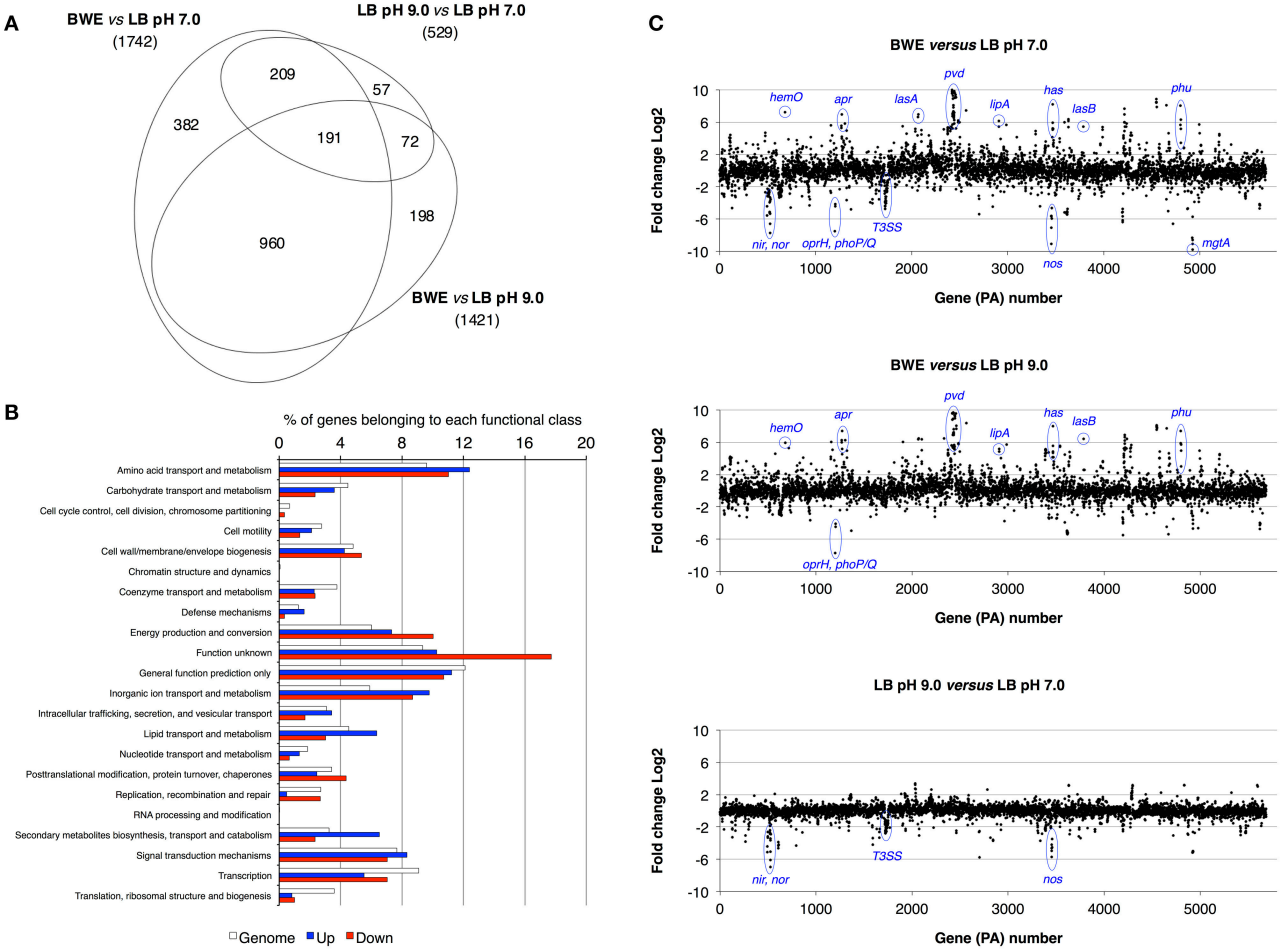
Characterization of *P. aeruginosa* gene expression. **(A)** Venn diagram showing the significantly differentially regulated genes (FDR < 5%) with a threshold fold change 2 (FC 2). Comparative expressions were performed for BWE vs. LB pH 7.0, BWE vs. LB pH 9.0, and LB pH 9.0 vs. LB pH 7.0. The elliptic Venn diagram was generated using eulerAPE software (Micallef and Rodgers, 2014). **(B)** Histograms representing the distribution of genes according their functional classes for *P. aeruginosa* whole genome (PAO1_genome) and for the up-regulated (Up) and down-regulated (Down) genes among the 960 differentially expressed both in BWE vs. LB pH 7.0 and pH 9.0. Data on the functional classes are from the *Pseudomonas* Genome Database (Winsor et al., 2016). **(C)** *P. aeruginosa* WT relative gene expression between BWE, LB pH 7.0, and LB pH 9.0 growth conditions. Gene annotation numbers are from the *Pseudomonas* Genome Database. Some significant up- and down-regulated genes discussed in the article are highlighted.

*P. aeruginosa* is an opportunistic pathogen causing infections in severe burn patient and represent a major problem for successful burn wound healing. While growing in burn wound exudate (BWE), *P. aeruginosa* was reported to induce the synthesis of several virulence factors (Gonzalez et al., 2016). To characterize in detail the complete transcription profile of *P. aeruginosa* while proliferating in BWE, a differential gene expression analysis was performed using the RNA-Seq approach (Supplementary Datasheet 1). RNA was extracted from bacterial cultures at an optical density (OD_600_) of two from three different growth conditions: a) human BWE, b) LB medium adjusted at pH 7.0 and c) LB at pH 9.0, corresponding to the BWE pH (Gonzalez et al., 2016). Transcriptomic results were further analyzed using the significant differentially expressed genes fold change (FC) ≥ 2 and false discovery rate (FDR) < 5% (Table 1). Differential gene expressions between BWE, LB pH 7.0 and LB pH 9.0 were depicted in a Venn diagram (Figure 1A and Supplementary Figure 1). These data showed that 960 genes were differentially regulated between BWE and both LB media, whereas expression of 529 genes was changing in LB pH 9.0 compared to LB pH 7.0. The 960 genes subgroup was divided into 620 up- and 340 down-regulated genes and their functional classes were indicated (Figure 1B). Representation of gene expression levels along the chromosome highlight the strong transcriptional remodeling occurring in BWE conditions compare to LB media (Figure 1C). Among the subgroup of 529 genes whose level is modified by the pH, 232 were up-regulated and 297 down-regulated genes. In order to validate the transcriptome analysis and to have a precise view of *P. aeruginosa* gene expression profiles over time, qRT-PCR were performed and expression levels were analyzed on a subset of genes whose functions were reported to contribute to *P. aeruginosa* pathogenesis. To this aim, RNA was extracted from cultures in BWE, LB pH 7.0 and LB pH 9.0 at OD_600_ 0.8, 1.1, 2.2, and 4.6. In BWE conditions, these OD_600_ values corresponded to 6, 12, 18, and 24 h of *P. aeruginosa* growth, respectively.

**Table 1:** Statistics of RNA-Seq data.

	**FDR > 5%**	**FDR < 5%**			
		**up**	**up FC2**	**down**	**down FC2**
BWE vs. LB pH 7.0	1111	2298	999	2139	743
			(18.0%)		(13.4%)
BWE vs. LB pH 9.0	1360	2126	864	2062	557
			(15.6%)		(10.0%)
LB pH 9.0 vs. pH 7.0	2300	1677	232	1571	297
			(4.2%)		(5.4%)

*Significant differentially expressed genes with false discovery rate (FDR) smaller than 5% and number of genes, which have a fold change (FC) 2 threshold. Both differentially up-regulated (up) and down-regulated (down) genes are indicated in the Table. The RNA-Seq analysis was performed on the full P. aeruginosa transcriptome (5548 transcripts) available*.

### Iron acquisition mechanism

**Figure 2:**
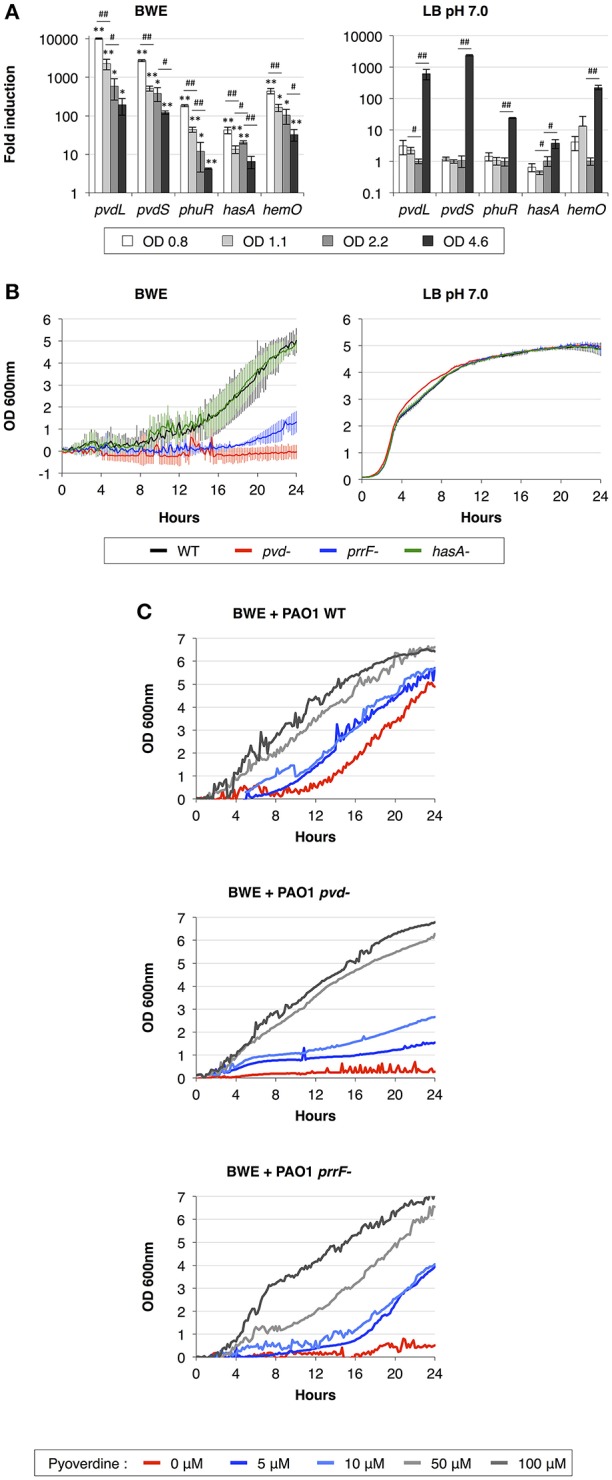
Importance of iron acquisition mechanisms for *P. aeruginosa* growth in BWE. **(A)** Gene expression kinetics of major mechanisms involved in iron uptake and acquisition were measured by qRT-PCR in BWE and LB pH 7.0 conditions at different OD_600_ values, as indicated. **(B)** Growth of *P. aeruginosa* WT and *pvd-, prrF*- and *hasA*-mutants was monitored by OD_600_ measurement in BWE and LB pH 7.0. **(C)** Growth kinetics of *P. aeruginosa* WT, *pvd*- and *prrF*-mutants in BWE supplemented with various concentrations of pyoverdine, as indicated. Statistics analyses are indicated with *P*-values. Comparison between similar time points in different culture media is indicated using ^*^P < 0.05 and ^**^P < 0.01. Comparison between times points in a same culture condition is indicated using ^#^*P* < 0.05 and ^##^*P* < 0.01.

During the infection process, *P. aeruginosa* uses several tools to acquire iron from the surrounding environment (reviewed in Cornelis, 2010). The iron acquisition mechanisms include the production of siderophores (reviewed in Mislin and Schalk, 2014), such as pyoverdine and pyochelin, and heme uptake mechanisms involving Phu and Has systems. The previous qRT-PCR analysis showed a strong induction of pyoverdine secretion in *P. aeruginosa* growing in human BWE (Gonzalez et al., 2016). This phenotype was further confirmed by RNA-Seq data showing an up-regulation of the entire pyoverdine biosynthetic pathway (Supplementary Table 3). Interestingly, *pvdL* and *pvdS* gene expression kinetics showed a very strong induction at early time points followed by a reduction in expression level at later growth phases (Figure 2A). The opposite dynamic was observed in LB media, where *pvdL* and *pvdS* genes showed weak expression prior to the induction at the stationary phase (Figure 2A). These data suggest that iron is weakly available for *P. aeruginosa* in BWE conditions whereas it becomes a limiting factor in LB only at later growth phases. To determine whether the pyoverdine machinery is required for *P. aeruginosa* development in BWE conditions, we monitored growth kinetics of the *pvd*-mutant and observed that this mutant was unable to proliferate in BWE, unlike in LB conditions (Figure 2B). Addition of pyoverdine to BWE was sufficient to restore growth phenotype of *pvd*-mutant strain in a dose-dependent manner. Pyoverdine supplementation to WT cultures could even reduce the lag period triggering a more rapid bacterial growth (Figure 2C).

We then decided to look at other iron acquisition mechanisms such as the heme uptake composed of *phu* genes (Ochsner et al., 2000) and heme oxygenase *hemO* (PA0672). RNA-Seq data showed an upregulation of the *phu* system and *hemO* expression in *P. aeruginosa* while proliferating in BWE compare to both LB growth conditions (Supplementary Table 3). This is consistent with the presence of hemoglobin in BWE as previously observed (Gonzalez et al., 2016). Kinetic expression analysis of the heme/hemoglobin uptake outer membrane receptor *phuR* (PA4710) and the heme oxygenase (PA0672) genes displayed similar profiles to *pvd* genes, meaning a strong initial induction followed by a gradual reduction of the expression level (Figure 2A). Expression of small RNAs *prrF1* (PA4704.1) and *prrF2* (PA4704.2) was strongly up-regulated in BWE (Supplementary Table 3). Located in the upstream region of *phu* operon, these two sRNA might be involved in *P. aeruginosa* iron homeostasis (Wilderman et al., 2004) and virulence (Reinhart et al., 2015). A higher level of these sRNA was observed in cystic fibrosis lung infection (Nguyen et al., 2014) and was reported to play a role in *P. aeruginosa* pathogenicity (Reinhart et al., 2015). To determine whether the regulatory activity mediated by sRNA *prrF* is important for *P. aeruginosa* growth in exudate, we generated a *prrF1/F2* mutant and monitored its growth kinetics in BWE. Interestingly, growth of the *prrF*-mutant was strongly affected in BWE whereas it appeared normal in LB pH 7.0 conditions (Figure 2B). The growth defect observed in the *prrF*-mutant can be restored by adding pyoverdine in the BWE medium suggesting that different iron acquisition mechanisms might complement each other (Figure 2C). Since *prrF* genes are located downstream of the *phu* operon, we investigated the putative polar effect caused by *prrF* deletion. A plasmid containing the PA4704.1 to PA4703.3 *prrF* region showed a recovery of *prrF*-growth profile similar to the corresponding WT control situation (Supplementary Figure 2A) and an OD_600nm_ significantly different from the uncomplemented *prrF*- strain (Supplementary Figure 2B). Interestingly, the presence of the construct containing *prrF* was affecting the WT growth, whereas the empty low copy plasmid did not. This result supports the fact *prrF* expression may be tightly regulated to allow optimal function.

Among the tools used to acquire iron, *P. aeruginosa* can also take advantage of the heme assimilation system Has (Ochsner et al., 2000), which is based on the secretion of the HasAp (PA3407) hemophore via a type I secretion system (T1SS) composed of *hasDEF* (PA3406-PA3404). The complete Has system, including the sensor *hasS* (PA3409) and the outer membrane receptor *hasR* (PA3408), was strongly up-regulated in BWE compare LB conditions (Supplementary Table 3). Expression kinetics of *hasAp* in BWE showed a high initial expression level that deceased in later time points (Figure 2A). In order to evaluate the role of the Has system in BWE condition a *hasAp* mutant was constructed. Analysis of growth kinetics revealed no difference between *P. aeruginosa* WT and *hasA*-mutant (Figure 2B), suggesting a non-essential function of the T1SS Has system for *P. aeruginosa* proliferation in BWE.

Taken together our results show an early and strong activation of iron acquisition mechanisms by *P. aeruginosa* growing in human BWE. These include production of siderophore and activation of the heme uptake and assimilation in combination with small RNA expression. Functional analysis of mutants deficient in iron uptake mechanism showed different growth profiles (Figure 2B), highlighting the essential function of *pvd* and *prrF* genes and suggesting that each of these systems may contribute differently to bacterial proliferation in BWE. Moreover, down-regulations of *bfrB* (PA3531) and *ftnA* (PA4235) encoding for bacterioferritin, involved in iron storage (Moore et al., 1986; Rivera, 2017), support the limited iron accessibility for *P. aeruginosa* in BWE (Supplementary Table 3).

### Quorum-sensing induction in BWE

**Figure 3:**
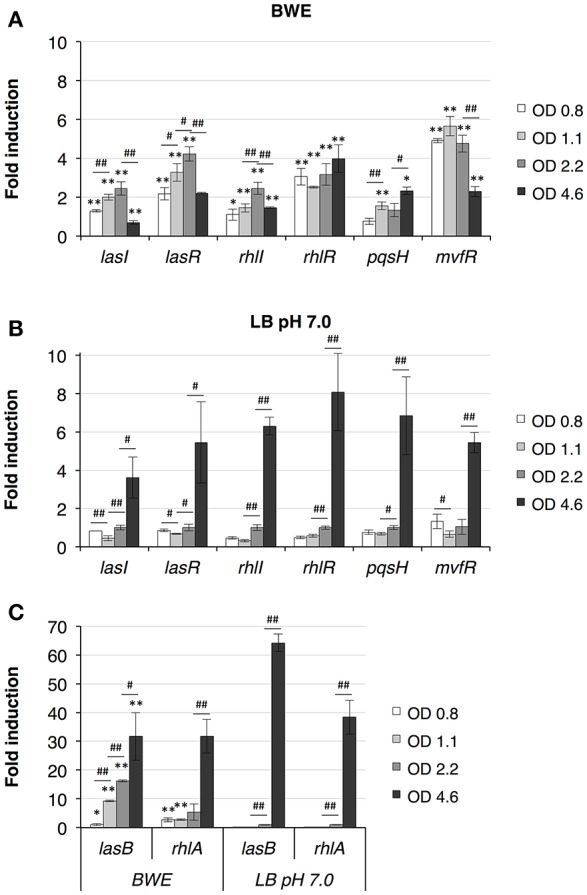
Quorum Sensing pathways induction in BWE. QS system activation were measured by qRT-PCR by following *lasI/R, rhlI/R* and *pqsH/mvfR* gene expression kinetics in BWE **(A)** and LB pH 7.0 **(B)** conditions at different OD_600_ as indicated. **(C)** Elastase (*lasB*) and gene involved in rhamnolipids synthesis (*rhlA*) were used to evaluate expression of virulence factors controlled by quorum sensing. Statistics analyses are indicated with *P*-values. Comparison between similar time points in different culture media is indicated using ^*^P < 0.05 and ^**^P < 0.01. Comparison between times points in a same culture condition is indicated using #*P* < 0.05 and ##*P* < 0.01.

*P. aeruginosa* virulence is mainly regulated by quorum sensing (QS) mechanisms. To determine whether QS was induced in BWE, we looked at the expression of the major QS systems, LasI/R, RhlI/R, PqsH/MvfR. As previously described (Gonzalez et al., 2016), up-regulation of the three main QS systems was only moderate in BWE at OD_600_ 2 compared to LB pH 7.0 and LB pH 9.0 conditions (Supplementary Table 3). However, when looking at expression kinetics of QS systems in BWE conditions up-regulation was observed already at early growth phases (Figure 3A) whereas it occurred only at later phases in LB 7.0 (Figure 3B). These data suggest that bacterial communication in BWE might be based on efficiency sensing (Hense et al., 2007) rather than a cell density-dependent mechanism. Importance of QS for bacterial growth inside BWE is further supported by the kinetics of *lasB* and *rhlA* expression displaying an up-regulation at OD 1.1 and OD 2.2 compare to similar densities in LB pH 7.0. These observations are consistent with an activation of QS pathways occurring in BWE already at early time points (Figure 3C).

### Secretion systems and multidrug efflux pumps

As described above, T1SS-Has was induced by *P. aeruginosa* in BWE but seemed not to be essential for the growth of bacteria. To determine whether the growth in BWE could impact on the regulation of other *P. aeruginosa* secretion systems, their expression profiles were analyzed.

#### Type I secretion systems (T1SS)

**Figure 4:**
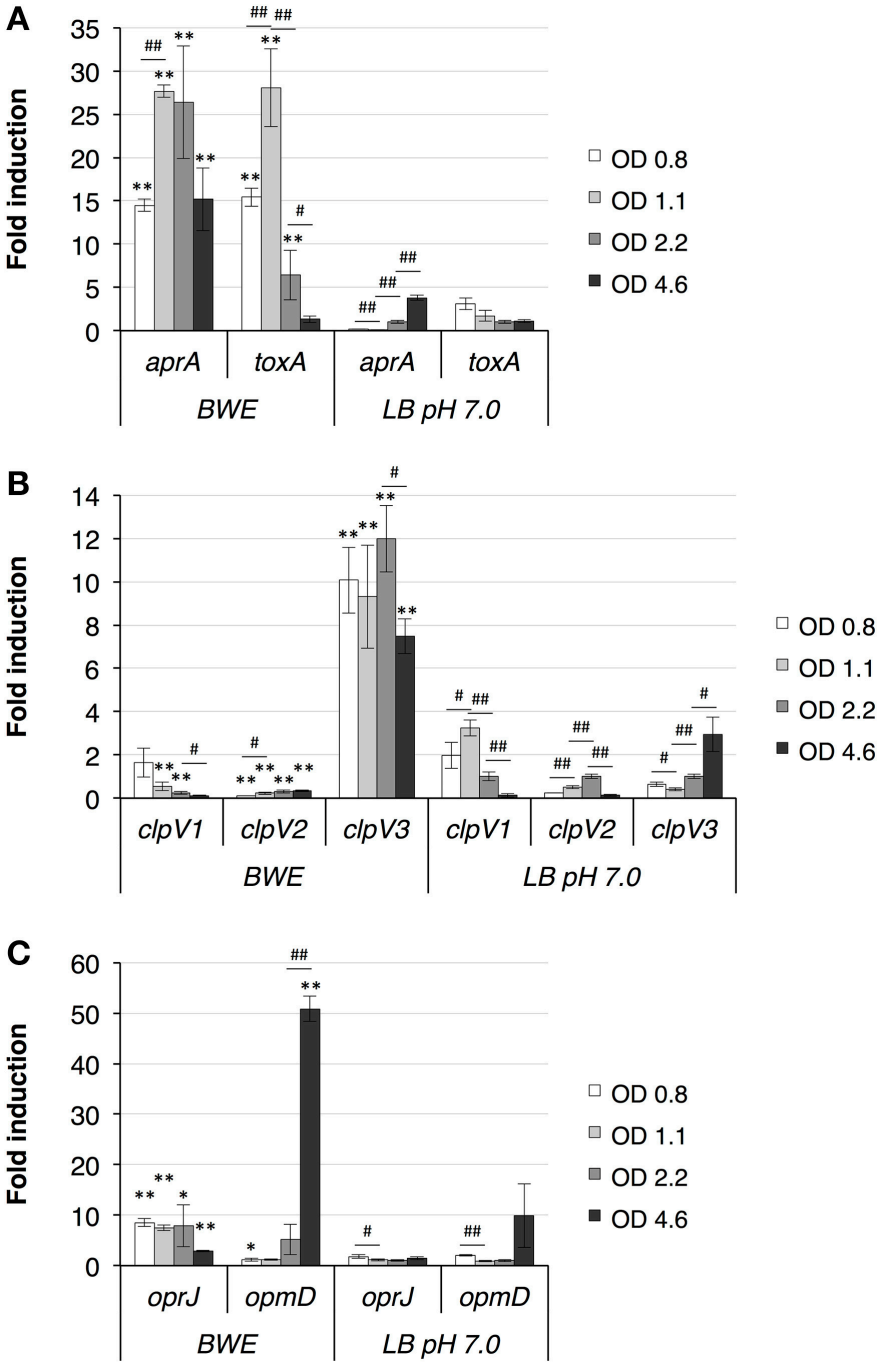
*P. aeruginosa* gene expression kinetics in BWE. Expression kinetics measured in BWE and LB pH 7.0 conditions at different OD_600_ values using qRT-PCR on the following genes: **(A)** alkaline protease (*aprA*) and exotoxin-A (*toxA*); **(B)** *clpV1, clpV2*, and *clpV3* ATPases for H1-, H2, and H3-T6SS, respectively; **(C)** *oprJ* for MexCD-OprJ system and *opmD* for MexGHI-OpmD. Statistics analyses are indicated with *P*-values. Comparison between similar time points in different culture media is indicated using ^*^P < 0.05 and ^**^P < 0.01. Comparison between times points in a same culture condition is indicated using #*P* < 0.05 and ##*P* < 0.01.

RNA-Seq data showed a strong induction of the Apr T1SS composed of *aprXDEFAI* (PA1245-PA1250) (Supplementary Table 3). The T1SS-Apr is responsible for the secretion of alkaline protease AprA (PA1249) and AprX (PA1245) (Duong et al., 2001). AprA, also known as serralysin, is reported to activate the epithelial sodium channel (ENaC) in airways leading to an increase in sodium absorption (Butterworth et al., 2012), whereas the function of AprX is still unknown. AprA was also reported to interfere with the host immunity by preventing pathogen recognition mechanisms via degradation of immunogenic flagellin molecules (Bardoel et al., 2011) and perturbation of complement pathway activation (Laarman et al., 2012). A recent report showed that, in addition to AprA, flagellin degradation was also mediated by the LasB protease (Casilag et al., 2016). In contrast to the Has system, which is regulated via the Fur protein, the Apr system contains a LasR binding site in the upstream region of *aprX* suggesting a QS-dependent regulation. In BWE, the *aprA* expression profile over time shows a maximum induction between 12 and 18 h (Figure 4A), which corresponds to the early growth phase of *P. aeruginosa* in BWE.

Two other probable T1SS were described in *P. aeruginosa*, PA1875-PA1877 and PA4142-PA4144 (Filloux, 2011). Both systems showed an increased expression in BWE conditions compared to LB control conditions (Supplementary Table 3). The PA1875-PA177 (*bapBCD*) system was shown to secrete PA1874 (*bapA*), which may function as a adhesion protein important during biofilm development (De Bentzmann et al., 2012). The substrate of PA4142-PA4144 is not yet identified, nevertheless, the PA4141 gene located upstream region displays some homology with the *E. coli* bacteriocin CvaC (Fath et al., 1994) and was shown to be up-regulated in BWE (Supplementary Table 3).

#### Type II secretion systems (T2SS)

T2SS are widely distributed among pathogenic bacteria and contribute to pathogenesis through the export of virulence factor proteins (reviewed in Korotkov et al., 2012). *P. aeruginosa* genome contains two T2SS, namely Xcp (Filloux et al., 1998), and Hxc (Ball et al., 2002). RNA-Seq data has shown a slight up-regulation in the transcription of all T2SS-Xcp encoding genes (Supplementary Table 3). The Xcp system is divided into two operons *xcpRSTUVWXYZ* (PA3103-PA3095) and *xcpPQ* (PA3104-PA3105) separated by a 219 bp region containing a LasR binding site that is responsible for the QS-dependent expression of T2SS-Xcp (Chapon-Herve et al., 1997). Among the virulence factors secreted by *P. aeruginosa* T2SS-Xcp (Filloux, 2011) a larger proportion showed higher expression levels in BWE compared to LB control conditions according to the RNA-Seq analysis (Supplementary Table 3). Secreted via T2SS-Xcp, the potent exotoxin-A (*toxA*) displayed an up-regulation already at low OD_600_ values (Figure 4A), Taken together these results showed an up-regulation of T2SS-Xcp structural components and secreted substrates, whereas expression level of the T2SS-Hxc machinery (PA0677-PA0687) and its substrate, alkaline phosphatase LapA (PA0688) (Ball et al., 2002), remained low (Supplementary Table 3).

#### Type III secretion system (T3SS)

*P. aeruginosa* virulence depends on the secretion of a large variety of active proteins (Filloux, 2011). The T3SS allows the secretion of effector proteins through a needle-like structure directly inside target host cells (Galle et al., 2012). RNA-Seq data has shown a down-regulation of all T3SS apparatus, regulators and effectors proteins in *P. aeruginosa* cultured in BWE and LB pH 9.0 compared to LB pH 7.0 (Supplementary Table 3). Taken together, transcription results suggest a lower gene expression of T3SS at basic pH compared to pH 7.0. These expression levels were confirmed using two components of the T3SS machinery, *pscU* (PA1690) and *pcrV* (PA1706), in order to monitor their transcription profile at different *P. aeruginosa* growth phases in BWE conditions (Supplementary Figure 3).

#### Type VI secretion system (T6SS)

The T6SS apparatus are central factor elements involved in microbial intra- and interspecies competition (reviewed in Russell et al., 2014). *P. aeruginosa* genome contains three T6SS (H1-, H2-, and H3-T6SS) (Mougous et al., 2006), (reviewed in Filloux et al., 2008; Sana et al., 2016). Transcription data of *P. aeruginosa* showed an up-regulation of the H3-T6SS gene clusters whereas H1- and H2-T6SS genes were more weakly expressed in BWE compared to both LB pH 7.0 and LB pH 9.0 (Supplementary Table 3). Analysis of *clpV3* gene expression kinetics confirmed the up-regulation of the H3-T6SS system at all tested time points (Figure 4B). Importantly, a role in eukaryotic epithelial cell internalization and *C. elegans* toxicity was observed in both QS-regulated H2-T6SS (Sana et al., 2012) and H3-T6SS (Sana et al., 2013).

#### Multidrug efflux (mex) pumps

Antibiotic resistance represents a major concern during wound infections especially in long-term therapy required for burn patients; therefore to determine whether BWE may trigger bacterial antibiotic resistance mechanisms, we analyzed the expression profiles of multidrug efflux systems and OprD porin whose repression is well known to confer carbapenem resistance (Trias and Nikaido, 1990). Results have shown an up-regulation of both *mexCD-oprJ* (PA4597-4599) and *mexGHI-opmD* (PA4205-PA4208) systems in the burn wound exudate conditions compared to both LB pH 7.0 and LB pH 9.0 control conditions (Supplementary Table 3). The MexCD-OprJ system was reported to increase resistance against chloramphenicol, cationic peptides, fluoroquinolones and tetracycline (reviewed in Fernandez and Hancock, 2012), whereas its induction can be triggered by bacteria membrane damaging agents such as chlorhexidine, a widely used clinical antiseptic (Morita et al., 2003; Fraud et al., 2008). The MexGHI-OpmD system was described to play an important role in AHL homeostasis (Aendekerk et al., 2002) and transport of phenazine molecules (Sakhtah et al., 2016). Expression of this system was almost abolished in *mvfR*-mutant, and showed also dependency on two others quorum sensing systems Las and Rhl (Deziel et al., 2005). Due to their different substrate affinities, we decided to analyze the gene expression profile of their respective outer membrane proteins OprJ and OpmD. qRT-PCR data displayed a rapid induction of *oprJ* expression in *P. aeruginosa* growing in BWE suggesting an immediate activation of membrane stress response, whereas *opmD* appeared to be strongly induced at later bacteria growth stages (Figure 4C) consistent with the observed QS activation kinetics (Figure 3A). Mex systems were described to contribute to antibiotic resistance and might negatively affect antibiotherapy. Furthermore, the absence of any strong down-regulation in *oprD* gene expression during *P. aeruginosa* proliferation in BWE may rule out carbapenem resistance induction (Supplementary Table 3).

### *P. aeruginosa* metabolism in BWE

#### Lactate and glucose metabolism

**Figure 5:**
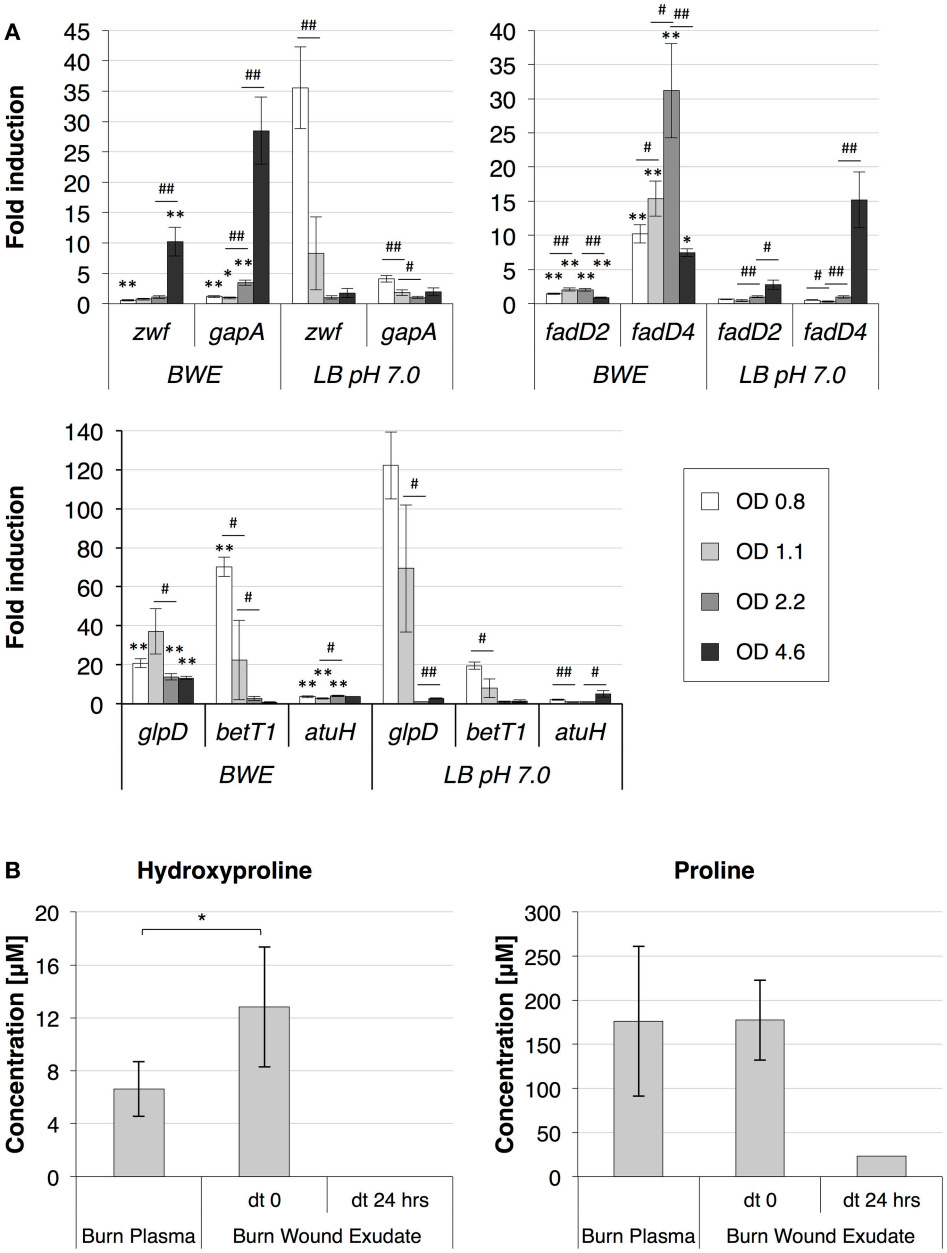
*P. aeruginosa* metabolism in BWE. **(A)** Expression profiles of glucose and lipid catabolic genes were measured over time by qRT-PCR in BWE and LB pH 7.0 conditions. Tested genes included glucose-6-phosphate dehydrogenase (*zwf*) and glyceraldehyde-3-phosphate dehydrogenase (*gapA*) (upper left panel), long-chain-fatty-acid-CoA ligases D2 (*fadD2*), and D4 (*fadD4*) (upper right panel), glycerol-3-phosphate dehydrogenase (*glpD*), choline transporter (*betT1*), and the long-chain-acyl CoA synthetase (*atuH*) (lower panel). Statistics analyses are indicated with *P*-values. Comparison between similar time points in different culture media is indicated using ^*^P < 0.05 and ^**^P < 0.01. Comparison between times points in a same culture condition is indicated using #*P* < 0.05 and ##*P* < 0.01. **(B)** The concentration of the hydroxyproline, a collagen degradation product, and proline were analyzed in burn patient plasma and BWE before and after 24 h of *P. aeruginosa* growth. Bacteria were removed by centrifugation (15,000 x g for 5 min) prior to chemical analysis of the culture supernatant. Statistics analyses are indicated using ^*^P < 0.05.

To further analyze *P. aeruginosa* physiology in a burn wound environment, the BWE chemical composition was determined before and after 24 h of bacterial growth (Table 2). Results have shown a full consumption of lactate (> 96%) by *P. aeruginosa*, whereas the glucose level decreased only by 10.3%. These data are consistent with *P. aeruginosa* carbon source preferences (Palmer et al., 2007). The low glucose consumption was further supported by the RNAseq data that showed no induction of the main genes involved in the glucose catabolism (Supplementary Table 3) (reviewed in Singh et al., 2016). Interestingly, despite stable expression of glucose porin *oprB* (PA3186), both glucose dehydrogenase *gcd* (PA2290) and gluconate permase *gnuT* (PA2322) showed induction in BWE compared to LB control conditions (Supplementary Table 3). These data suggest that *P. aeruginosa* stimulates the pathway involved in gluconate production, whereas glucose catabolism, via glucose-6-P and 2-keto-6P-gluconate branches, as well as glycolysis, were repressed. Recent reports have highlighted the strong variation in gluconate secretion by *P. putida* depending on iron levels (Sasnow et al., 2016). Interestingly, in BWE genes involved in the glucose catabolism, such glucose-6-phosphate dehydrogenase *zwf* (PA3183) and glyceraldhehyde-3-phosphate dehydrogenase *gapA* (PA3195), showed an induction only after 24 h growth, suggesting that other carbon source are preferred at earlier growth phases (Figure 5A upper left panel). An opposite kinetics was measured in LB conditions with an early induction of glucose catabolism followed by a decrease in gene expression at later growth phases (Figure 5A upper left panel).

**Table 2:** BWE chemical composition after *P. aeruginosa* growth.

**Component**	**Unit**	**BWE dt0**	**BWE + PAO1 24h**	**Variation (%)**
Fe	μM	9	7	−22
Lactate	mM	5.1	<0.20	> −96
Glucose	mM	5.8	5.2	−10.3
${\text{PO}}_{4}^{3-}$	mM	1.1	0.6	−44.3
Mg^2+^	mM	0.8	0.6	−18.4
Triglycerides	mM	0.6	<0.1	> −83.3
Cholesterol	mM	1.2	0.4	−66.7
Proteins	g/L	34	33	−2.9
Ca^2+^	mM	1.4	1.5	6.4
Urea	mM	5.7	8.2	43.9
${\text{NH}}_{4}^{+}$	μM	166	4,530	2,629
Pyruvate	μM	177	205	15.8

*BWE composition was analyzed before and after 24 h of P. aeruginosa growth. Negative and positive variations correspond, respectively, to the percentage of component consumed and produced in the medium. A positive variation can be due to bacterial biosynthesis or the breakdown of BWE components*.

#### Lipid metabolism

Analysis of the lipid concentrations in BWE after 24 h of *P. aeruginosa* growth showed a drop in triglycerides (−83%) and cholesterol (−67%) (Table 2). These observations might be linked to the up-regulation of several lipases (Supplementary Table 3). During infection, *P. aeruginosa* was reported to use other types of lipids such as phosphatidylcholine (PC) (Sun et al., 2014). PC, which is one of the most abundant lipids in human fluids, can be degraded by *P. aeruginosa* into phosphorylcholine head group, glycerol and long chain fatty acids. These elements are further catabolized by choline (Bet), glycerol (Glp), and fatty acid degradation (Fad) pathways (Zarzycki-Siek et al., 2013; Sun et al., 2014). In order to gain a better insight into *P. aeruginosa* lipid metabolism within BWE, we looked at the expression profiles of these different lipid catabolic pathways (Supplementary Table 3). RNA-Seq data showed a general up-regulation of *P. aeruginosa bet, glp*, and *fad* gene families. Expression kinetics analysis of these pathways showed an induction of *fadD2, fadD4* and *betT1* genes during early growth phases in BWE whereas up-regulation appeared later or at lower level in LB control conditions, supporting a preferential utilization of lipids by *P. aeruginosa* while growing in BWE (Figure 5A upper right and lower panels). The long-chain-acyl CoA synthetase (*atuH*) involved acyclic terpenes utilization was also more expressed in BWE compare to LB control conditions (Figure 5A lower panel). Taken together these observations confirm the importance of nutrient sources, and in particular the lipid catabolism, during *P. aeruginosa* development in BWE.

#### Protein metabolism

The protein concentration in BWE showed similar levels with burn human plasma suggesting a BWE protein content mainly composed of serum albumin and enriched with collagen residues released at the wound site of the burn patient. In addition, during colonization *P. aeruginosa* can degrade collagen molecules via the secretion of elastase and alkaline protease AprA (Bejarano et al., 1989). The collagen breakdown releases large amounts of the modified amino acid hydroxyproline (Hyp) (Gorres and Raines, 2010), which can be further catabolized by *P. aeruginosa lhp* genes (Li and Lu, 2016). Analysis of BWE composition revealed a higher Hyp concentration than in burn patient plasma, consistent with a possible Hyp release at the burn wound site caused by basal lamina destruction (Figure 5B). Interestingly, the up-regulation of Hyp catabolic genes observed in the RNA-Seq data (Supplementary Table 3) was coupled with the full reduction of Hyp amount in BWE after 24 h of *P. aeruginosa* growth (Figure 5B). Analysis of proline concentration in BWE conditions also showed a strong reduction (−87%) consistent with the highest carbon preferences measured in Cystic Fibrosis (CF) sputum (Palmer et al., 2007). This observation was further supported by the up-regulation of the proline porin *opdB* (PA2700) in BWE compared to LB control conditions (Supplementary Table 3). Since proline is not a collagen degradation product, its concentration was similar in burn plasma and BWE (Figure 5B). Taken together our data highlight the capacity of *P. aeruginosa* to produce both collagen and hydroxyproline degrading enzymes. Activation of bacterial protein catabolism might be further supported by the massive increase in ammonium (+2628%) and urea (+44%) concentrations measured in BWE after 24 h of *P. aeruginosa* growth (Table 2). Taken together our results revealed, that despite a profound remodeling of *P. aeruginosa* physiology in BWE, no changes in expression were observed in motility, adhesion or alginate production (Supplementary Table 3). Moreover, no differences in morphology were observed by optical microscope between BWE and LB control conditions (not shown).

### BWE stimulate expression of non-ribosomal peptides synthetases (NRPS)

In addition to the translational machinery, bacteria also use a non-ribosomal mediated mechanisms to synthetize a wide range of complex molecules (Challis and Naismith, 2004), like pyoverdine siderophores (Calcott et al., 2014). Interestingly, RNA-Seq data showed an up-regulation of most NRPS identified gene clusters (Gulick, 2017) compared to both LB control conditions (Supplementary Table 3). Among *P. aeruginosa* NRPS, some are of high interest especially in the context of host-microbe interactions. This is the case for the *ambABCDE* (PA2306-PA2302) cluster encoding for enzymes involved in L-2-Amino-4-methoxy-*trans*-3-butenoic acid (AMB) synthesis (Lee et al., 2010; Rojas Murcia et al., 2015). The AMB, a γ-substituted vinylglycines, was described to be a potent antibiotic and toxin produced by *P. aeruginosa* (Lee et al., 2010) causing amoebal encystment in salt buffer and triggering a dose-dependent growth inhibition in rich medium (Lee et al., 2012). Analysis of *ambB* expression in *P. aeruginosa* cultured in BWE displayed a higher level compare to LB conditions already at early time points (Supplementary Figure 4). AMB was also reported to block *Erwinia amylovora* growth and to act as a weak seed germination arrest factor in *Poa annua* (Lee et al., 2013).

## Discussion

This study provides for the first time a global view of *P. aeruginosa* gene expression profile in human burn wound exudate (BWE) growth conditions. Our data highlights the specific transcriptional pattern produced by *P. aeruginosa* and provides new insight in the physiology of this pathogen in the context of human burn wound infections. Moreover, this study allows a better understanding of the temporal activation of metabolic pathways necessary for *P. aeruginosa* development. Therefore, taken together, the data effectively raises possibilities to develop new strategies to treat bacterial infections in the context of severe burn patients.

**Figure 6:**
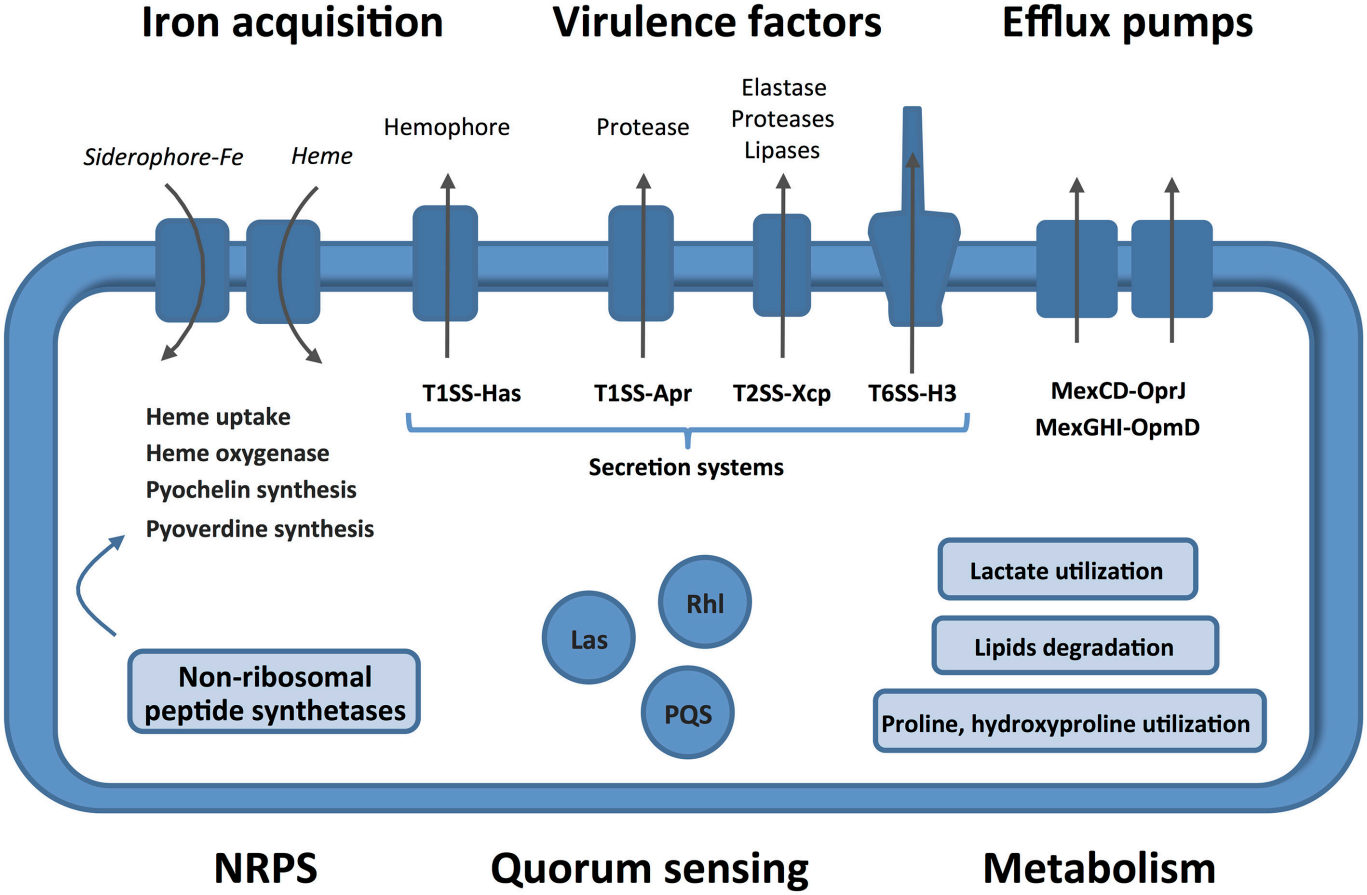
Features and characteristics of *P. aeruginosa* growing in human BWE. Details are given in the text. The activated pathways are shown in the entire figure. These characteristics uncover therapeutic approaches to combat the development of *P. aeruginosa* on wounds of burn victims.

Human burn wounds are characterized by the production of significant amounts of BWE creating a particular environment that require particular physiological properties from bacteria to proliferate. Our analysis showed that *P. aeruginosa* were inducing profound physiological changes to adapt to these specific human BWE growth conditions (Figure 6). We confirmed in BWE, both by chemical and gene expression analyses, the *P. aeruginosa* physiological preference for lactate instead of glucose that was previously described in CF condition (Palmer et al., 2007). Our transcriptomic analysis also highlighted the activation of a range of lipid catabolic pathways indicating that lipids may be an important source of carbon and energy during bacterial development in human BWE. These results are further supported with data coming from burned animal model experimentation showing a role of long-chain fatty acid degradation during the *P. aeruginosa* infection process (Turner et al., 2014). Fatty acid degradation was also reported to be necessary for successful lung tissue colonization and infection in mice (Kang et al., 2010). Another characteristic of burn wounds is the presence of a large quantity of damaged tissue. These tissue residues mainly composed of collagen and found in the BWE, represent a major source of nutrient for the colonization of pathogenic bacteria. We showed herein that both hydroxyproline, release from collagen molecules, and proline were extensively catabolized by *P. aeruginosa*. Moreover, a dramatic down-regulation of antioxidant capacities was observed after burn injury (Horton, 2003). Expression of the glutathione *S*-transferase A4 (GSTA4), involved in the conjugation of the lipid peroxidation byproduct 4HNE, was decreasing in muscle and fat tissues whereas an up-regulation was only measured in skin (Apidianakis et al., 2012). Interestingly, a negative correlation could be established between the GSTA4 expression level and the development of bacterial infections both in human and mice. The capacity of *P. aeruginosa* to adjust optimally its physiology to BWE, by activating lipid and collagen catabolism, may explain its remarkable efficiency to infect burn wounds and proliferate in BWE, which could not be observed for other tested pathogens (Gonzalez et al., 2016).

One of the major limiting-factors for bacteria during host infection is often the low iron availability. To counteract this, *P. aeruginosa* can rely on several iron uptake and acquisition mechanisms. Our data showed a very rapid and strong induction of these mechanisms by *P. aeruginosa* while growing in human BWE. This includes activation of pyoverdine siderophores and heme uptake machineries. Interestingly, deletion mutants for iron uptake were strongly affected in their development inside BWE. For example, *pvd*-mutant, unable to produce pyoverdine, could not show growth in BWE, whereas addition of siderophore was sufficient to restore proliferation. More interestingly, pyoverdine addition could even reduce the lag period observed in *P. aeruginosa* WT suggesting that prior to proliferation bacteria need to produce and secreted some iron-chelating molecule into the BWE. Our data further highlight that despite similarities in gene expression kinetics, each iron acquisition mechanism may contribute differentially to bacterial growth in BWE. New therapeutic approaches, known as Trojan horse strategies, are currently developed. Their aim is to stimulate bacteria uptake of antibiotics via iron uptake pathways by attaching these molecules to siderophores (Mislin and Schalk, 2014; Gasser et al., 2016). The strong activation of iron acquisition mechanisms by *P. aeruginosa* growing in BWE emphasizes interesting perspectives for these future strategies of severe burn wound infections.

Bacterial populations control the infection process using a form of communication, called Quorum Sensing (QS), which plays a crucial role in coordination of bacterial behavior. Complex environments, such as BWE, influence both the spatial distribution of bacteria populations and the diffusion parameters of QS autoinducer molecules. These conditions favor a spatial heterogeneity challenging the classical bacteria density-dependent communication model. Bacterial growth in complex environments can give rise to the concept of “efficient sensing,” which integrates elements such as bacteria cell density but also QS autoinducer molecules diffusion and stability (Hense et al., 2007). Despite maximum QS activation during later growth periods, our data have highlighted an early activation of QS pathways in BWE compared to LB conditions, which suggests that QS activation in burn wounds may not exclusively correlate with bacteria cell density. Nevertheless, currently developed strategies based on anti-QS approaches portray interesting perspectives in a context of burn wound infections (Gupta et al., 2015), for which bacteria grow heterogeneously and induce virulence factor production already at low OD values.

Global gene expression analysis of *P. aeruginosa* growing in BWE revealed an activation of a broad range of virulence factors including lipases, toxins and proteases, which are representative of a *P. aeruginosa* acute infection pattern (Turner et al., 2014). Interestingly, *P. aeruginosa* modified the expression of secretory machineries such as the T1SS-Apr, responsible for the secretion of alkaline protease AprA, or the T2SS-Xcp, involved in the release of different virulence factors whose expression were also up-regulated. Taken together these observations support an increased capacity of *P. aeruginosa* to provoke overall damage to host tissues. Among the induced secretion machineries, the H3-T6SS is of particular interest with a possible role in eukaryotic host–microbe interaction during host invasion (Sana et al., 2016). Dynamic and regulation of this interaction may contribute to explain *P. aeruginosa* capacity to proliferate inside human BWE. In addition, H3-T6SS effector PA2374 was described to play a function in iron acquisition mechanism via secreted outer-membrane vesicles (OMVs) as recently reported (Lin et al., 2017). On the other hand, the well-described T3SS showed only a weak expression in *P. aeruginosa* proliferating in BWE. This absence of induction is surprising since T3SS is currently considered as a marker of acute infection (Turner et al., 2014). The BWE environment must therefore trigger a more complex regulation of the secretion system. Moreover, *pcrV* was used to produce antibodies with positive effect against *P. aeruginosa* infections in the burn mice model (Holder et al., 2001). Our observation, therefore, represents an important point in the adaptation of animal research to human medicine. Antibodies against T3SS component may not be optimal targets in the context of human burn wound infection by *P. aeruginosa*. Description of *P. aeruginosa* physiology and genome expression in BWE provides new insights for the clinical understanding of burn wound infections and appears to be essential to develop new and efficient strategies against pathogenic microorganisms.

## Author contributions

MG, BF, PJ, LA, WR, Y-AQ, and KP: designed the study; MG, VD, SL, and BF performed the experiments; MG, Y-AQ, and KP: analyzed the data; MG and KP: wrote the paper. All authors reviewed the results and approved the final version of the manuscript.

### Conflict of interest statement

The authors declare that the research was conducted in the absence of any commercial or financial relationships that could be construed as a potential conflict of interest.

## Supplementary material

Supplementary Figure 1 Venn diagram of differentially expressed genes. Distribution of significantly (FDR < 5) differentially expressed (fold change > 2) genes between BWE and LB pH 7.0 growth conditions. Venn diagrams depict up- and down-regulated genes **(A)**, up-regulated genes **(B)** and down regulated genes **(C)**.

Supplementary Figure 2 Complementation of *prrF*-mutant in BWE. Histogram showing culture OD_600nm_ at 24 h of *P. aeruginosa* PAO1 WT and *prrF*-mutant transformed with pME6001, as a control, and pME6001-prrF for complementation. Bacteria were cultured in BWE and OD_600nm_ were measured over time **(A)** and statistically analyzed at 24 h **(B)**. Statistics analyses are indicated using ^*^*P* < 0.05 and ^**^*P* < 0.01.

Supplementary Figure 3 *P. aeruginosa* T3SS expression in BWE. Gene expression kinetics of T3SS components *pcrV* and *pscU* were measured by qRT-PCR in BWE and LB pH 7.0 conditions at different OD_600_ values. Comparison between similar time points in different culture media is indicated using ^*^*P* < 0.05 and ^**^*P* < 0.01. Comparison between times points in a same culture condition is indicated using #*P* < 0.05 and ##*P* < 0.01.

Supplementary Figure 4 *P. aeruginosa* AMB expression in BWE. Gene expression kinetics of the non-ribosomal peptide synthetase involved in AMB biosynthesis was measured by qRT-PCR in BWE and LB pH 7.0 conditions at different OD_600_ values. Comparison between similar time points in different culture media is indicated using ^*^*P* < 0.05 and^**^*P* < 0.01. Comparison between times points in a same culture condition is indicated using #*P* < 0.05 and ##*P* < 0.01.

Supplementary Table 1 **(A)** Bacteria strains and plasmids used in this study. **(B)** List of primers used to generate deletion mutants. **(C)** Primers used for gene cloning.

Supplementary Table 2 Primers used for qRT-PCR.

Supplementary Table 3 Differential gene expression depending on growth condition measured by RNA-seq.

Supplementary Datasheet 1 Raw data.
