# Supplementary figures and images for: Transcriptome Analysis of Pseudomonas aeruginosa Cultured in Human Burn Wound Exudates
Source: Front Cell Infect Microbiol. 2018 Feb 27;8:39. doi: 10.3389/fcimb.2018.00039 (PMC5835353; doi:10.3389/fcimb.2018.00039)

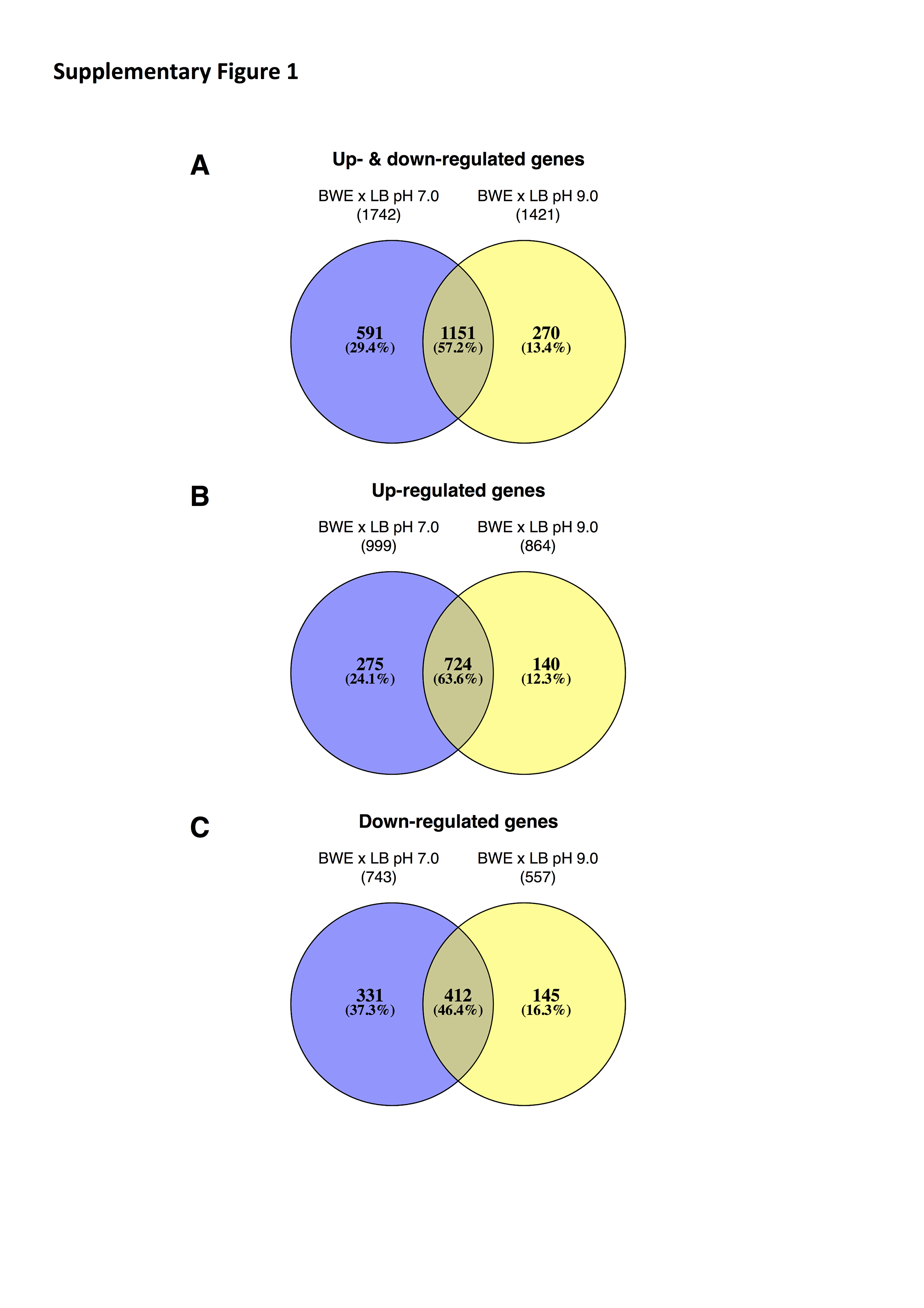

Supplement: Supplementary Figure 1 — Venn diagram of differentially expressed genes. Distribution of significantly (FDR < 5) differentially expressed (fold change > 2) genes between BWE and LB pH 7.0 growth conditions. Venn diagrams depict up- and down-regulated genes (A), up-regulated genes (B) and down regulated genes (C). [file Image1.TIFF]

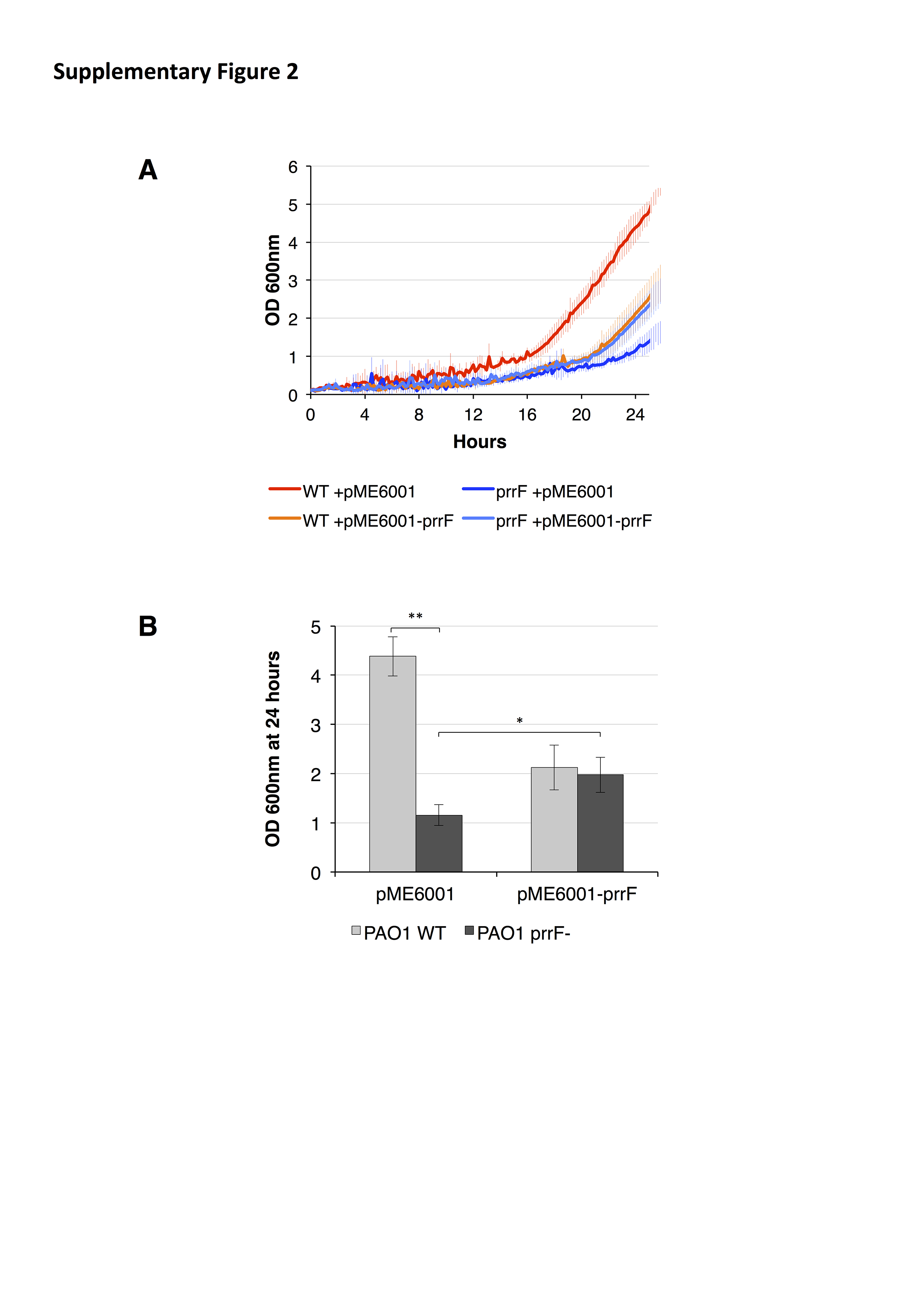

Supplement: Supplementary Figure 2 — Complementation of prrF-mutant in BWE. Histogram showing culture OD600nm at 24 h of P. aeruginosa PAO1 WT and prrF-mutant transformed with pME6001, as a control, and pME6001-prrF for complementation. Bacteria were cultured in BWE and OD600nm were measured over time (A) and statistically analyzed at 24 h (B). Statistics analyses are indicated using *P < 0.05 and **P < 0.01. [file Image2.TIFF]

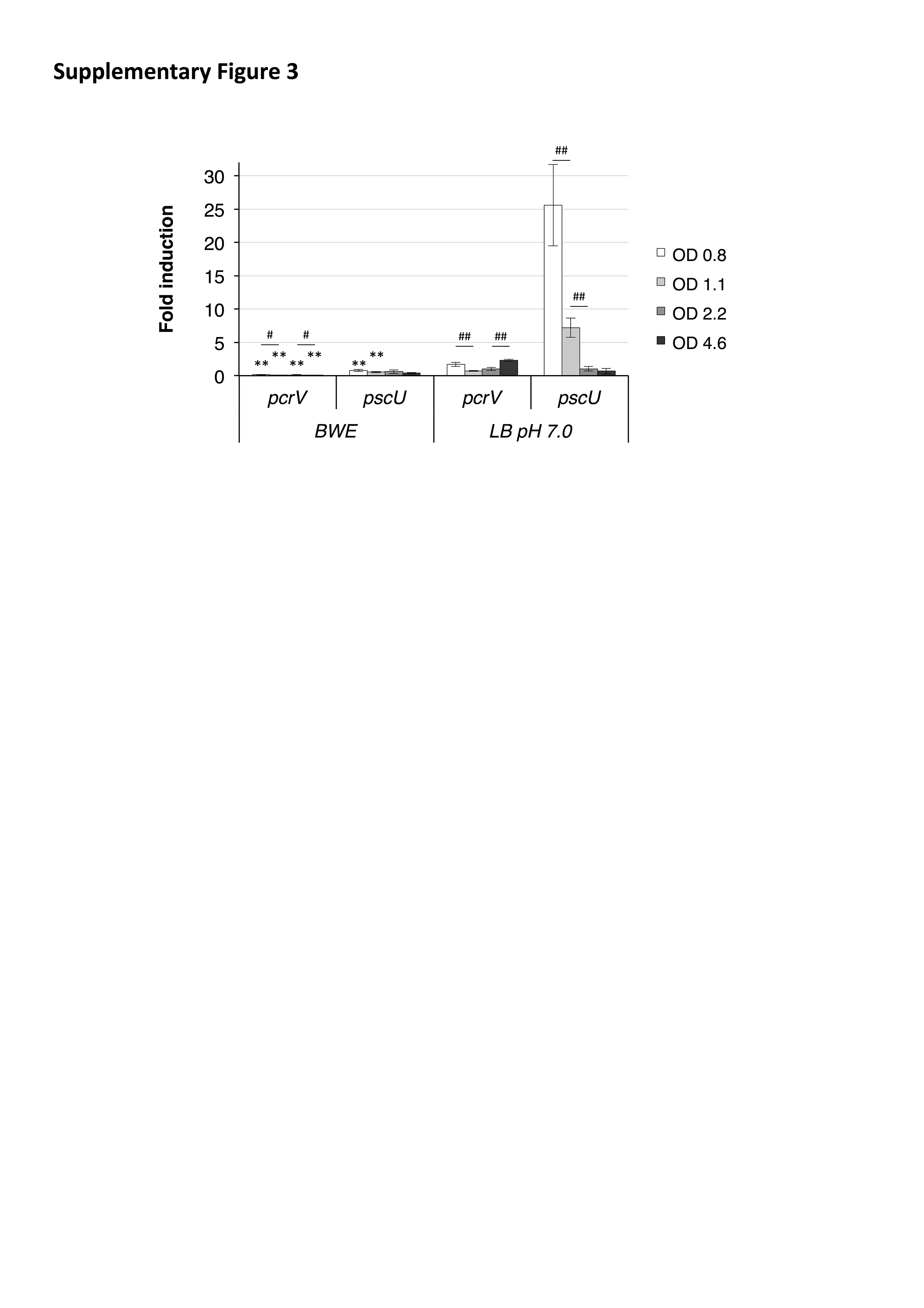

Supplement: Supplementary Figure 3 — P. aeruginosa T3SS expression in BWE. Gene expression kinetics of T3SS components pcrV and pscU were measured by qRT-PCR in BWE and LB pH 7.0 conditions at different OD600 values. Comparison between similar time points in different culture media is indicated using *P < 0.05 and **P < 0.01. Comparison between times points in a same culture condition is indicated using #P < 0.05 and ##P < 0.01. [file Image3.TIFF]

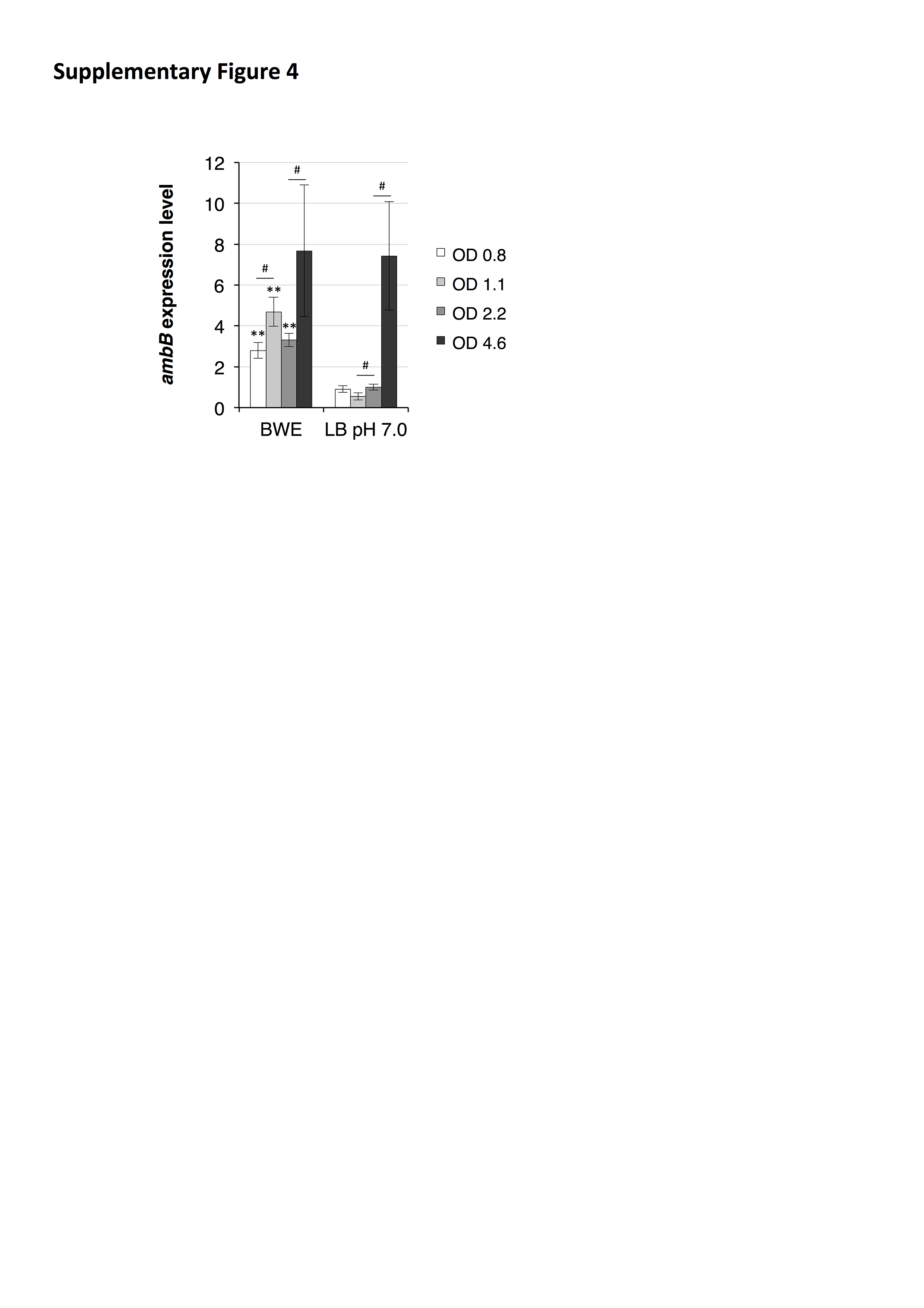

Supplement: Supplementary Figure 4 — P. aeruginosa AMB expression in BWE. Gene expression kinetics of the non-ribosomal peptide synthetase involved in AMB biosynthesis was measured by qRT-PCR in BWE and LB pH 7.0 conditions at different OD600 values. Comparison between similar time points in different culture media is indicated using *P < 0.05 and**P < 0.01. Comparison between times points in a same culture condition is indicated using #P < 0.05 and ##P < 0.01. [file Image4.TIFF]
