# Supplementary material for: Transcriptome Analysis of Pseudomonas aeruginosa Cultured in Human Burn Wound Exudates
Source: Front Cell Infect Microbiol. 2018 Feb 27;8:39. doi: 10.3389/fcimb.2018.00039 (PMC5835353; doi:10.3389/fcimb.2018.00039)
Supplement: Supplementary Table 1 — (A) Bacteria strains and plasmids used in this study. (B) List of primers used to generate deletion mutants. (C) Primers used for gene cloning. [file Table1.PDF]

Supplementary Table 1

A. Bacteria strains and plasmids used in this study.

Strain	Genotype	Reference
P. aeruginosa		
PAO1	PAO1; *P. aeruginosa* PT5 wild-type	
PALS128-3	PAO1; *pvd-*	(1)
PAO1 *ΔhasA*	PAO1; *hasA-*	This study
PAO1 *ΔprrF1/F2*	PAO1; *prrF-*	This study
E.coli		
DH5α	rec*A1*, end*A1*, hsd*R17*, deo*R*, thi-*1*, sup*E44*, gyr*A96*, rel*A1*, *Δ*(lac*ZYA*-arg*F*), *U169*(φ80dlac*Z*ΔM15)	(2)
TOP10	*F-mcrA Δ(mrr-hsdRMS-mcrBC) Φ80lacZΔM15 Δ lacX74 recA1 araD139 Δ(araleu)7697 galU galK rpsL(Str^R) endA1 nupG*	Invitrogen
Plasmid		
pME3087	*Suicide plasmid*	(3)
pME6001	*Cloning vector; Gm^r*	(4)
pME6001-*prrF*	pME6001 containing *prrF1/F2 region*	This study

B. List of primers used to generate deletion mutants.

Deletion mutant		Primers sequences
hasA	*part A*	attcgagctcggtacccgggCGGTCGCCGCCCGCCGGT
		acgccggccgGCCAAAACTCCAATTCAACGAGTGGTGAATGCCGGAG
	part B	gagttttggcCGGCCGGCGTCGCCCATG
		cctgcaggtcgactctagagAGGTAGAGGCCGAGCACCATGAGG
prrF	*part A*	attcgagctcggtacccgggGCGATGCGCTGGCGCACC
		caaagtgccgTCTCTCAGCTTACCGGCTGATCTCG
	part B	agctgagagaCGGCACTTTGCCGGGTCT
		cctgcaggtcgactctagagAGCGGAGCGAGATCATGC

C. Primers used for gene cloning.

Gene mutant	Primer name	Primers sequences
prrF	*prrF_F*	GCGggatccTAGCCTGATCGAAGGCATGG
	prrF_R	CGGaagcttTATCGCGCTACGTTTTCGCG

- 1. Visca P, Serino L, & Orsi N (1992) Isolation and characterization of Pseudomonas aeruginosa mutants blocked in the synthesis of pyoverdin. *J Bacteriol* 174(17):5727-5731.
- 2. Sambrook JF, E. F.; Maniatis, T. (2001) Molecular Cloning: A Laboratory Manual (Third Edition) Cold Spring Harbor, NY, USA. *Cold Spring Harbor Laboratory Press*.
- 3. Wenner N, Maes A, Cotado-Sampayo M, & Lapouge K (2014) NrsZ: a novel, processed, nitrogen-dependent, small non-coding RNA that regulates Pseudomonas aeruginosa PAO1 virulence. *Environ Microbiol* 16(4):1053-1068.
- 4. Blumer C, Heeb S, Pessi G, & Haas D (1999) Global GacA-steered control of cyanide and exoprotease production in Pseudomonas fluorescens involves specific ribosome binding sites. *Proc Natl Acad Sci U S A* 96(24):14073-14078.
