# Supplementary material for: Transcriptome Analysis of Pseudomonas aeruginosa Cultured in Human Burn Wound Exudates
Source: Front Cell Infect Microbiol. 2018 Feb 27;8:39. doi: 10.3389/fcimb.2018.00039 (PMC5835353; doi:10.3389/fcimb.2018.00039)
Supplement: Supplementary Table 2 — Primers used for qRT-PCR. [file Table2.PDF]

Supplementary Table 2

Gene_name	Gene_ID	Primer_name	Primer_sequence
ambB	PA2305	*ambB_F*	ACGAGATCAGCCTGCTCAAT
		ambB_R	CGTCGAATAGGTGGTGTCCT
aprA	PA1249	*aprA_F*	CTGACCAAACCGAACGACTT
		aprA_R	GCGTCGACGAAGTGGATATT
atuH	PA2893	*atuH_F*	AAGGGCTTCATGGAGAAGGT
		atuH_R	AACTGGGTGTGCTTGAAACC
betT1	PA5375	*betT1_F*	CCTCATCCTCTATCCCGACA
		betT1_R	GCGAAGTAGAGCAGCGAGAT
clpV1	PA0090	*clpV1_F*	TGTCGAGGAAGCGGTGGAGCG
		clpV1_R	ACCTTGAGCTTGGCGAATTCCG
clpV2	PA1662	*clpV2_F*	CGCGGGGCGAGCACAGCGCCTC
		clpV2_R	GCGCAACGGGTTGCGTAGCAGG
clpV3	PA2371	*clpV3_F*	GGAGCTGTTCAAGGCCGGCAAT
		clpV3_R	TCGCGGGAGGCCTGGATCCACTCC
fadD2	PA3300	*fadD2_F*	TGGCGAACTCTGTGTGAAAG
		fadD2_R	TCGATCTCGTTGGGGTAGAC
fadD4	PA1617	*fadD4_F*	TTCCAGATGGGGGTGTATTC
		fadD4_R	TTCTCGGTCATCCCGTAGAC
gapA	PA3195	*gapA_F*	ACACCAACGACCAGAACCTC
		gapA_R	CCTCGTCTACGCTGGTATCC
glpD	PA3584	*glpD_F*	ATCCAGGACGACCTGAAACA
		glpD_R	AGGTAGGCGGTTTCCTCTTC
hasAp	PA3407	*hasAp_F*	AGGAGGTGAGCTTCAGCAAC
		hasAp_R	AGGTGGAGTTGATCGACAGG
hemO	PA0672	*hasAP_F*	ACCTCTTCCAGCATGACCTG
		hasAP_R	GAACCTTCGGAAACGAACAG
lasB	PA3724	*lasB_F*	AAGCCATCACCGAAGTCAAG
		lasB_R	GTAGACCAGTTGGGCGATGT
lasI	PA1432	*lasI_F*	CTACAGCCTGCAGAACGACA
		lasI_R	ATCTGGGTCTTGGCATTGAG
lasR	PA1430	*lasR_F*	ACGCTCAAGTGGAAAATTGG
		lasR_R	GTAGATGGACGGTTCCCAGA
mvfR	PA1003	*mvfR_F*	AACCTGGAAATCGACCTGTG
		mvfR_R	TGAAATCGTCGAGCAGTACG
opmD	PA4208	*opmD_F*	CCTGGTGGAGTTTCTTCGAC
		opmD_R	CGTCGTAGTCCAGCTGTTGT
oprF	PA1717	*oprF_F*	GGTTACTTCCTGACCGACGA
		oprF_R	TCGCTGTTGATGTTGGTGAT
oprJ	PA4597	*oprJ_F*	GTTCCGGGCCTGAATGCCGCTGC
		oprJ_R	TCGCGGCTGACCAGGGTCTGACG
pcrV	PA1706	*pcrV_F*	TTCCTGGTGTCGGCCTATTT
		pcrV_R	CTGGATCACGCTGTAGACCT

pqsH	PA2587	*pqsH_F*	ATGTCTACGCGACCCTGAAG
		pqsH_R	AACTCCTCGAGGTCGTTGTG
pscU	PA1690	*pscU_F*	GTACATCGACCTGCCGTTTC
		pscU_R	GATCTTCTTCAGGTCCGGCT
pvdL	PA2424	*pvdL _F*	ACCCTGCGTGCTGATGTC
		pvdL _R	TCGGCTCGGAACCGGAGAA
pvdS	PA2426	*pvdS_F*	AGATCACTTCGTCGTTCAAGGCA
		pvdS_R	GATGTGTTCGAGGGTCGCGTA
rhlA	PA3479	*rhlA_F*	CGAGGTCAATCACCTGGTCT
		rhlA_R	GACGGTCTCGTTGAGCAGAT
rhlI	PA3476	*rhlI_F*	CTCTCTGAATCGCTGGAAGG
		rhlI_R	GACGTCCTTGAGCAGGTAGG
rhlR	PA3477	*rhlR_F*	AGGAATGACGGAGGCTTTTT
		rhlR_R	CCCGTAGTTCTGCATCTGGT
toxA	PA1148	*toxA_F*	TGCTGCACTACTCCATGGTC
		toxA_R	CGTGGATGAACACCTTGATG
zwf	PA3183	*zwf_F*	GCAGATCGTCATCCACTTCA
		zwf_R	CGTGGTAGGTCTCGGAAAAA
